# Magnetic shielding properties of an iron-based nanocrystalline alloy for induction heating systems

**DOI:** 10.1016/j.heliyon.2024.e37119

**Published:** 2024-08-30

**Authors:** Feng Li, Ruifeng Zhao, Yibo Liu, Yang Xiao, Peng Sun, Jiamao Luo, Jun Wen, Zhihong Chen, Jing Hu, Zuqiang Qi

**Affiliations:** a China Tobacco Guang Dong Industrial Co., Ltd, Guangzhou, 510385, China; b Shenzhen First Union Technology Co., Ltd, Shenzhen, 518101, China

**Keywords:** Nanocrystalline alloy, Magnetic shielding films, Fragmentation degree, Induction heating

## Abstract

A nanocrystalline alloy, with an iron-based composition (Fe_58.5_Si_16.7_B_6.5_Nb_5.1_Cu_13.2_) and a Curie temperature of 570 °C, was investigated for its effectiveness as magnetic shielding films in an induction heating system. The primary focus of the research was to evaluate the shielding performance of the 3-turned (9-layered) shielding films with dimensions of 135 mm × 17 mm × 0.15 mm. Upon winding, these films formed a cylindrical structure that enveloped the coil, with a diameter of 13.9 mm and a height of 17 mm. The results showed that increasing the degree of fragmentation within the nanocrystalline shielding films significantly reduced the magnetic permeability by decreasing the real component from 11,500 to 400 and the imaginary part from 2800 to 20. However, a lower degree of fragmentation led to a 10 % increase in the resistance (*Rs*) of the heating module, although this effect was less pronounced as the relative permeability continued to increase. Furthermore, observations on preheating time to a set temperature of 400 °C and total energy consumption over a duration of 250s revealed an initial downward trend, followed by a rapid increase that even exceeded the initial values as the magnetic permeability of the nanocrystalline shielding films augmented. Notably, the study emphasized that nanocrystalline shielding films with a relative permeability value of 1000 demonstrated exceptional magnetic shielding performance, resulting in a 12.5 % reduction in preheat time and 7 % less energy consumption during preheating. In addition to empirical findings, the study developed a theoretical model elucidating the shielding mechanism inherent in induction heating systems. This model serves as a robust framework for the application of nanocrystalline shielding materials in such systems, laying the groundwork for enhanced magnetic shielding capabilities in future applications.

## Highlights

• Exploring iron-based nanocrystalline alloy shielding film for induction heating applications.
• Accessing impact of fragmentation on permeability and shielding performance of nanocrystalline films.
• Formulating a theoretical model to elucidate shielding mechanisms of nanocrystalline films in induction heating.
• Outlining future strategies to enhance shielding performance in induction heating systems.

## 1 Introduction

The rapid development of electronic products and wireless communication technology [1] has led to a surge in electromagnetic pollution, causing equipment interference, potential electromagnetic information leakage, and threats to human health [[2], [3], [4], [5]]. The practice of electromagnetic wave shielding involves employing shielding structures to restrict the propagation of electromagnetic waves in a given space. The various forms of electromagnetic interference (EMI) shielding can be categorized into three distinct types: electric field shielding [6], magnetic field shielding [7,8], and electromagnetic field shielding [9,10]. Electric field shielding uses a hollow conductor to prevent external electric fields from entering a protected area. Magnetic field shielding focuses on preventing external magnetic fields from interfering with equipment by using materials with high magnetic permeability. Electromagnetic field shielding targets high-frequency electromagnetic wave interference and relies on conductive or soft magnetic materials to block and absorb high-frequency electromagnetic waves. Researchers have extensively explored electromagnetic shielding materials for various applications. Gao et al. [[11], [12], [13]] specially investigated carbon nanotubes (CNT) for these purposes. They incorporated silicon-wrapped ammonium polyphosphate (SiAPP)/multi-wall carbon nanotubes (MWCNT) into electroconductive polystyrene (PS) through balling mill and hot-pressing techniques, creating a segregated structure that improved flame retardant properties and EMI shielding effectiveness [11]. Another study discovered that incorporating titanium carbide (Ti_3_C_2_T_x_) and cetyltrimethyl ammonium bromide-multi-wall carbon nanotubes (C-MWCNT) into PS composites significantly enhanced fire retardancy, thermal stability, and electromagnetic shielding capabilities [12]. Gao et al. [13] also developed gram-scale B/N co-doped porous carbon nanosheets (PCNs) with excellent microwave absorption performance, showing that the intrinsic electromagnetic wave absorption performance of these materials could be adjusted by varying the size of nanosheets and heteroatoms [13]. Zhang et al. [14] developed a sustainable bio-inspired dual-network material featuring mono-dispersed ANFs and SWCNTs, exhibiting a high fracture strength, adjustable electrical conductivity, remarkable EMI shielding effectiveness, and superior Joule heating performance with fast thermal response and wide temperature range.

Induction heating is a non-contact heating method known for its rapid and high-intensity heating generation and remarkable electric-to-heat conversion efficiency. It finds applications in various fields such as brazing, hardening, annealing, soldering, and even in 4H-SiC crystal growth [[15], [16], [17]] for the next generation of power devices and epitaxial graphene on Si-terminated SiC surface [[18], [19]]. However, the issue of electromagnetic pollution caused by induction heating systems is often disregarded or overlooked. In particular, heated tobacco products (HTPs), which can significantly reduce harmful constituents [20], suffer from electromagnetic pollution caused by their inner induction heating units. To minimize interference from metallic shell, mitigate electromagnetic pollution, and improve induction heating efficiency, appropriate shielding materials should be employed to isolate the magnetic fields generated by induction heating units.

Traditional EMI shielding materials primarily focus on electromagnetic field shielding, regulating the transmission of electromagnetic waves [[21], [22], [23], [24], [25], [26]]. The overall shielding efficiency is determined by a combination of reflection losses, absorption losses, and multiple reflection losses [27,28]. To analyze the shielding efficiency, a vector network analyzer can be utilized [1]. Extensive studies on EMI shielding mechanisms propose models that highlight reflections and absorptions as the main contributors to shielding efficiencies [1]. However, since induction heating operates at frequencies ranging from power frequency to several tens of MHz [29], while the skin effect of low-frequency electromagnetic waves is negligible, resulting in minimal absorption loss, and the wave impedance for these waves remains minimal, leading to slight reflection loss. Consequently, the magnetic field shielding effect plays a dominant role.

Moreover, the shielding material serves a different purpose in induction heating devices—it is wrapped around inductor coil to redirect the electromagnetic field towards the heating chamber, acting as an electromagnetic flux concentrator. The primary objective of the shielding material in this case is to enhance heat generation in the heater, also known as a susceptor. Typically, the susceptor is made of a soft magnetic alloy with high permeability and conductivity. Additionally, without shielding material, the inductor in an induction heating device can induce eddy currents and hysteresis losses in its adjacent components. Therefore, in the context of induction heating device, minimizing the transmission of the magnetic energy from the system and absorption losses of the introduced magnetic shielding materials is crucial. While some applications have been patented on magnetic shielding for induction heating [30], limited work has been dedicated to understanding its mechanism.

Soft magnetic materials, such as silicon steel [31], ferrite [32,33], permalloy [34,35], iron-based amorphous alloys [36,37], and iron-based nanocrystalline alloys, possess desirable properties for effective electromagnetic shielding, including high saturation magnetization strength, low coercivity, and minimal iron loss. Ferrite is widely used due to its low cost, high saturation flux density, and low magnetic loss [[38], [39]]. However, it has relative low permeability and low Curie temperature, limiting its applications in high-temperature environments. Iron-based content nanocrystalline alloys, categorized into four systems like FINEMET [40,41], NANOPERM [42,43], HIPERM [44,45] and NANOMET [46,47], exhibit excellent soft magnetic properties [48,49], including high permeability and excellent high-temperature stability [50].

To overcome the limitations of nanocrystalline alloys, including high electrical conductivity and increased eddy current losses in induction heating applications, the fragmentation process of nanocrystalline alloy ribbons has been explored [51]. This process involves mechanically crushing multilayered ribbons bonded together with polymer adhesive tape into smaller fragments, effectively suppressing overall eddy current losses by confining ring-shaped eddy currents to smaller alloy fragments and increasing the overall resistivity of the material.

This study focuses on investigating the impact of the fragmentation degree on the magnetic shielding properties of an iron-based nanocrystalline alloy with the composition of Fe_58.5_Si_16.7_B_6.5_Nb_5.1_Cu_13.2_. The magnetic shielding properties were evaluated by analyzing the preheating time to 400 °C and power consumption of induction heating devices utilizing various multilayer nanocrystalline shielding films. The selection of an appropriate shielding material is crucial for minimizing energy losses and maximizing heating efficiency. A theoretical model is proposed to enhance the understanding of the shielding mechanism in induction heating systems. The experimental data, theoretical analysis, and model are invaluable in enhancing the understanding of induction heating shielding mechanisms, enabling improvements in efficiency, reduction of magnetic radiation, and offering potential applications across various fields.

## 2 Experimental procedures

### 2.1 Specimen

Table 1 presents six specimens of Fe_58.5_Si_16.7_B_6.5_Nb_5.1_Cu_13.2_ nanocrystalline alloy, purchased from Advanced Technology & Materials Co., Ltd. The relative permeability values provided by the supplier correspond to the testing results at a frequency of 13.56 MHz. The nanocrystalline alloy ribbons were produced using a planar flow casting process with rapid solidification, where the roll speed was 30 m/s, and the cooling rate was 10^6^ °C/s. Subsequently, the ribbons underwent annealing at 550 °C for 60 min to relieve stress and transform the amorphous alloy into nanocrystal. The initial electrical resistivity is 1.2*10^−6^ Ω·m, with a saturation induction density of 1.25 T. This material possesses a Curie temperature of 570 °C, rendering it suitable for a range of high-temperature applications. To fabricate a composite magnetic shielding film, three layers of nanocrystalline ribbons were bonded together using an acrylic ester adhesive. Each layer had a thickness of 20 μm, and the resulting 3-layered shielding film had a total thickness of 0.15 mm, inclusive of an additional polyethylene terephthalate (PET) protecting film and double-sided tape for affixing the shielding films in various applications. Following bonding, the composite magnetic sheet underwent a mechanical fragmentation process to achieve varying levels of magnetic permeability. This process involved grinding the nanocrystalline sheets between a pair of rollers, with the option for multiple grinding cycles to further refine the degree of fragmentation and attain diverse magnetic shielding performance.

**Table 1:** Specimen information.

Specimen number	Relative Permeability (at 13.56 MHz)	Number of layers	Single layer thickness (μm)
AN400	400	3	20
AN700	700	3	20
AN1000	1000	3	20
AN3000	3000	3	20
AN5000	5000	3	20
AN8000	8000	3	20

The high magnetic permeability of the un-fragmented shield film results in substantial eddy current losses. Employing a multilayer strip structure is intended to boost the fragmentation effect and reduce eddy current losses. During the fragmentation process, the large-area continuous nanocrystalline structure was fragmented into smaller pieces through mechanical crushing. Prior to fragmentation, toroidal eddy currents were present within the ribbons but became confined within much smaller fragments after fragmentation. These smaller fragments were electrically isolated by the cracks and adhesive, effectively decreasing eddy currents.

### 2.2 Composition and microstructure test

The microstructure and composition of the nanocrystalline alloys were characterized using X-ray diffraction (XRD, Bruker D8 Advance) with Cu Ka radiation and X-ray photoelectron spectroscopy (XPS, PHI 5000 Versa Probe II). The morphology of the nanocrystalline alloy ribbons with varying degrees of fragmentation was observed using a metallographic microscope (KEYENCE VHX-6000) and a scanning electron microscope (SEM, NeoScope JCM-7000).

### 2.3 Magnetic shielding property test

The complex permeability of the specimens was measured using a soft magnetic measuring instrument (TUNKIA TS4000) following the ASTM D5568-22 standard. The measurements were conducted at a working frequency of 275 kHz, and the results were expressed as: *μ* = *μ*' – j*μ''*, where, *μ* represents the permeability of the sample, *μ′* stands for the real part of *μ*, reflecting the energy storage parameter, and *μ''* denotes the imaginary part of *μ*, indicating the power dissipation or loss parameter.

**Fig. 1:**
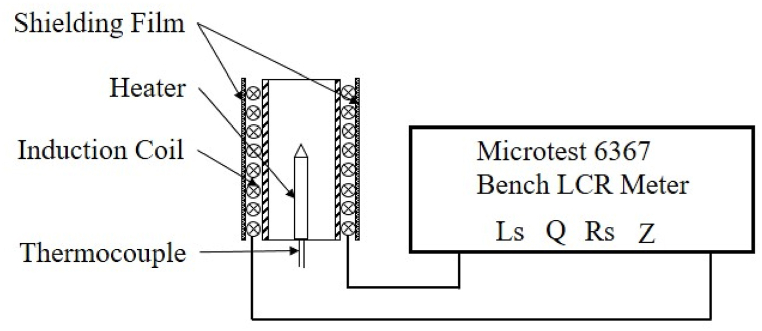
Schematic diagram of LCR testing. A benchtop LCR meter assesses *L*_*S*_, *Q*, *R*_*S*_, and *Z* parameters for an induction heating unit. The ends of the induction coil connect to the LCR meter electrodes for testing, with a frequency of 275 kHz aligning with application needs.

The impedance parameters of a spiral induction coil with various nanocrystalline shielding films were measured using a Microtest 6367 Bench LCR meter, as depicted in Fig. 1. The measured parameters encompassed inductance (*L*_*S*_), quality factor (*Q*), resistance (*R*_*S*_), and impedance (*Z*). The coil is made up of 300 strands of Litz wires, each with a diameter of 0.03 mm, tightly wound around a polyetheretherketone (PEEK) support in 15 turns to form a spiral coil. The testing frequency was set at 275 kHz to match the steady working frequency of the HTP heating device. To completely cover the coil, the height of the shielding film surpasses that of the coil, and its length extends beyond the coil's circumference with a specific overlap. For a standard shielding film wrapping around a coil with 3 turns, the dimensions are 135 mm × 17 mm × 0.15 mm, and the cylinder film has a diameter of 13.9 mm and a height of 17 mm. These shielding films served as flux concentrators, redirecting the exterior magnetic field flux towards the coil, reducing the penetration of magnetic field flux through the exterior shielding films, and preventing magnetic losses, magnetic interference, or contamination of nearby objects.

**Fig. 2:**
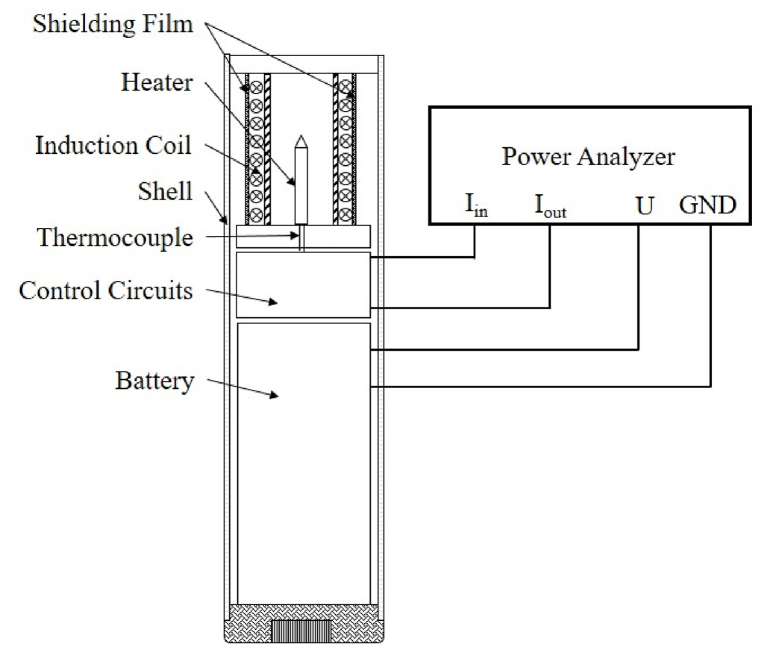
Schematic diagram of an induction heating device and energy consumption testing. Preheating energy consumption and total energy consumption for the heating device were gauged using a power analyzer. The analyzer connects to the Li-ion battery, with current in series and voltage in parallel configuration.

Fig. 2 illustrates the schematic diagram of a HTP induction heating device and energy consumption testing. It features a needle-shaped soft magnetic alloy pin at the center, acting as the heater, a spiral induction coil, shielding films, a metal-oxide-semiconductor field-effect transistor (MOSFET) in the control circuits for frequency control and current supply, a Li-ion battery, and an aluminum alloy shell. The battery's voltage output is transformed into a square wave voltage by utilizing MOSFET AC switching for more precision and control in the voltage generation process. This voltage then passes through the coil, capacitor, and returns to the battery's negative terminal to form a closed-loop circuit. By controlling the MOSFET switch through software programming, a square wave with a frequency of 275 kHz is generated. This current, after passing through a series LC resonance circuit, generates heat in the heater. A thermalcouple connected to the heater provides real-time temperature feedback to ensure adherence to predefined operating conditions. Temperature response curves of devices with different magnetic shielding films were recorded using a thermalcouple meter (GRAPHTECH midi Logger GL240) by attaching a K-type thermalcouple to the heater surface.

Energy consumption testing was carried out using a Yokogawa WT310E power analyzer, connecting current in series and voltage in parallel with the Li-ion battery. The HTP device operated within a temperature range of 350–400 °C, set at a target 400 °C for comparison. The shielding efficiencies of the nanocrystalline alloy films were evaluated by comparing heating rates and energy consumption. In induction HTP devices, heating occurs on the heater located inside the coil. The heater, composed of a soft magnetic alloy, has significantly higher permeability than the surrounding air gap or PEEK structural components within the coil. Consequently, most of the magnetic field flux within the spiral coil is drawn to the heater, where it is converted into thermal energy through eddy current heating and hysteresis loss heating.

## 3 Results and discussion

### 3.1 Structure of Fe_58.5_Si_16.7_B_6.5_Nb_5.1_Cu_13.2_ nanocrystalline alloy

**Fig. 3:**
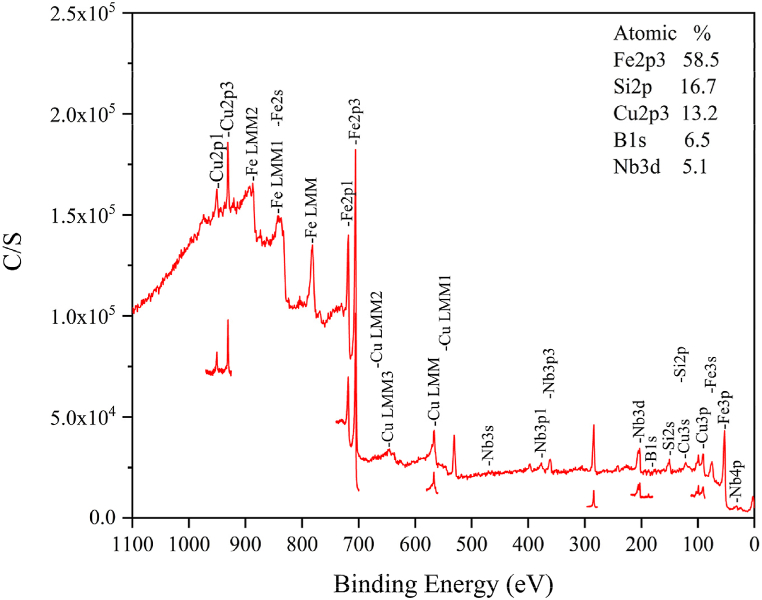
XPS spectra of a nanocrystalline alloy sample. XPS analysis carried out to ascertain the elemental composition of a nanocrystalline alloy sample. Before data collection, a 30-s etching process was employed to remove surface contaminants. The figure presents atomic percentage data for each element.

Fig. 3 displays the XPS spectra of the nanocrystalline alloy. Before collecting the elemental data, the sample underwent a 30-s etching process to eliminate surface contamination. The figure provides atomic percentage information for each element present in the alloy, as outlined in Table 2. The composition includes Fe_58.5_Si_16.7_B_6.5_Nb_5.1_Cu_13.2_, with iron content exceeding 50 %.

**Table 2:** Composition of a nanocrystalline alloy sample.

Element	At%
Fe 2p3	58.5
Si 2p	16.7
Cu 2p3	13.2
B 1s	6.5
Nb 3d	5.1

**Fig. 4:**
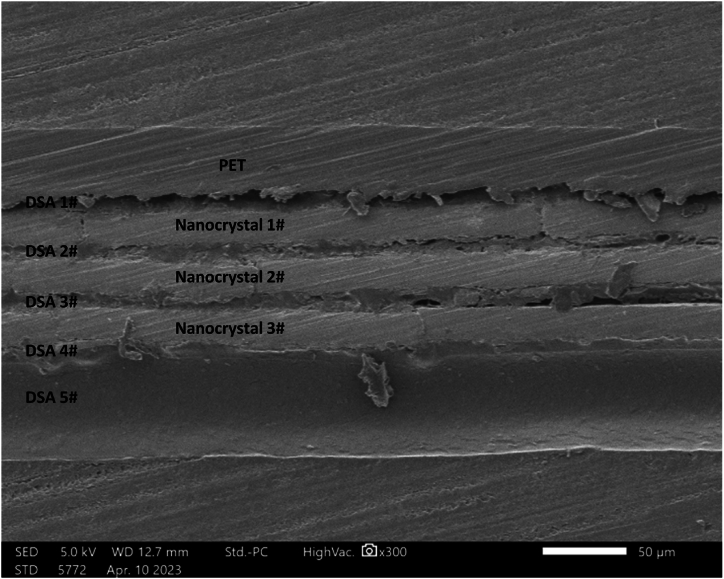
SEM morphology of a nanocrystalline alloy shielding film. The film comprises three nanocrystalline layers, each 20 μm thick, bonded with adhesive. The top layer is shielded by a 30 μm PET film, while the bottom layer is attached with a 50 μm double-sided adhesive.

Fig. 4 illustrates the SEM morphology of a nanocrystalline alloy shielding film, exhibiting a layered structure. The film comprises three nanocrystalline layers (Nanocrystal 1#∼3#), each with a thickness of 20 μm, that are bonded together using an adhesive (DSA 1#∼4#). The top layer is shielded with a 30 μm PET film, while the bottom of the alloy layer is attached with a double-sided adhesive (DSA5#), which has a thickness of 50 μm.

**Fig. 5:**
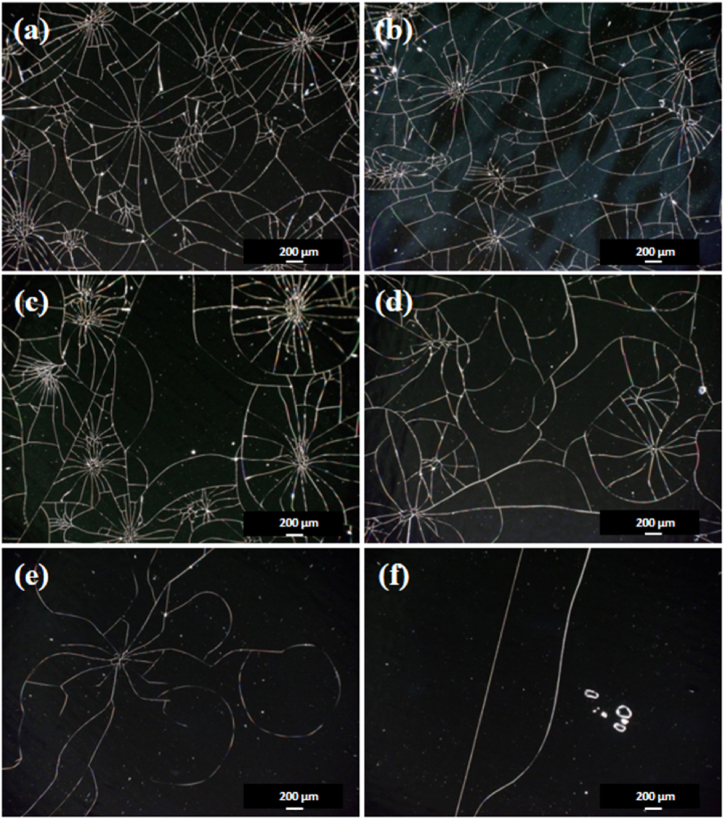
Metallographic microscope images of AN400(a), AN700(b), AN1000(c), AN3000(d), AN5000(e), and AN8000(f). Metallurgical microscopy unveils that increased fragmentation of nanocrystalline alloys results in diminished magnetic permeability. Smaller macroscopic sub-block sizes lead to lower magnetic permeability by fostering the creation of extra air gaps within the alloy, impeding the flow of magnetic flux.

Metallurgical microscopy analysis was conducted on nanocrystalline alloy shielding films with varying degrees of fragmentation to investigate the underlying mechanism behind their soft magnetic properties (as depicted in Fig. 5). The study unveiled that a decrease in the average macroscopic sub-block size corresponded to a reduction in magnetic permeability. This suggests an inverse correlation between the degree of fragmentation and magnetic permeability. Essentially, heightened fragmentation leads to the creation of more air gaps within the nanocrystalline alloy, hindering the connection of magnetic flux and thereby diminishing magnetic permeability.

**Fig. 6:**
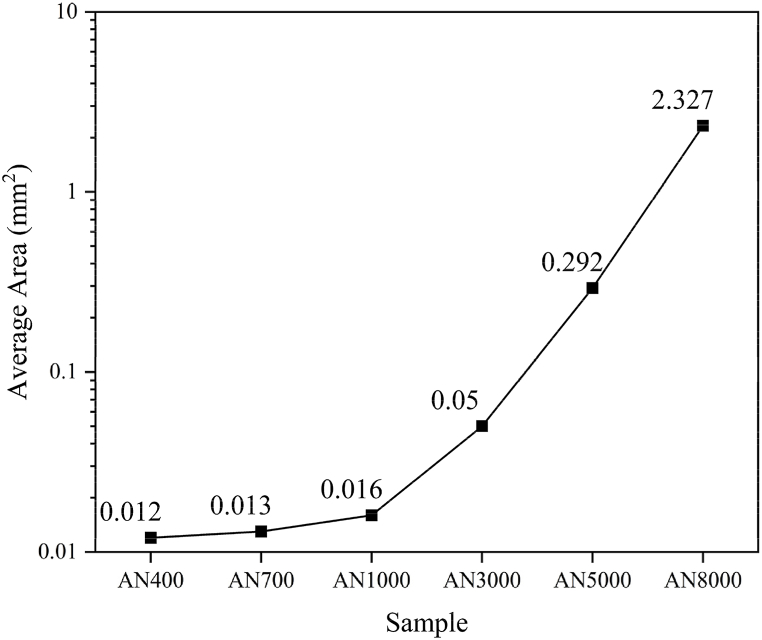
Analysis of fragmentation block area in nanocrystalline alloy with varying magnetic permeability. A larger average block area signifies a lower fragmentation degree, defined by fewer and larger fragmented blocks in the alloy film. This decreased fragmentation is associated with higher magnetic permeability as larger fragmented blocks offer smoother paths for magnetic flux and enhanced magnetic coupling. Thus, a positive relationship is observed between the area of fragmented blocks and magnetic permeability in nanocrystalline alloy shielding films.

Fig. 6 illustrates the correlation between the average area of fragmentation block regions and magnetic permeability in nanocrystalline alloy. The average area spans from 0.012 to 2.327 mm^2^, reflecting diverse degrees of fragmentation and permeability. A larger average area indicates a lower degree of fragmentation, implying fewer and larger fragmented blocks in the alloy film. This decreased fragmentation is linked to higher magnetic permeability. The existence of larger fragmented blocks facilitates smoother paths for magnetic flux and improved magnetic coupling, leading to enhanced permeability. Hence, there exists a positive relationship between the area of fragmented blocks and magnetic permeability in nanocrystalline alloy shielding films.

**Fig. 7:**
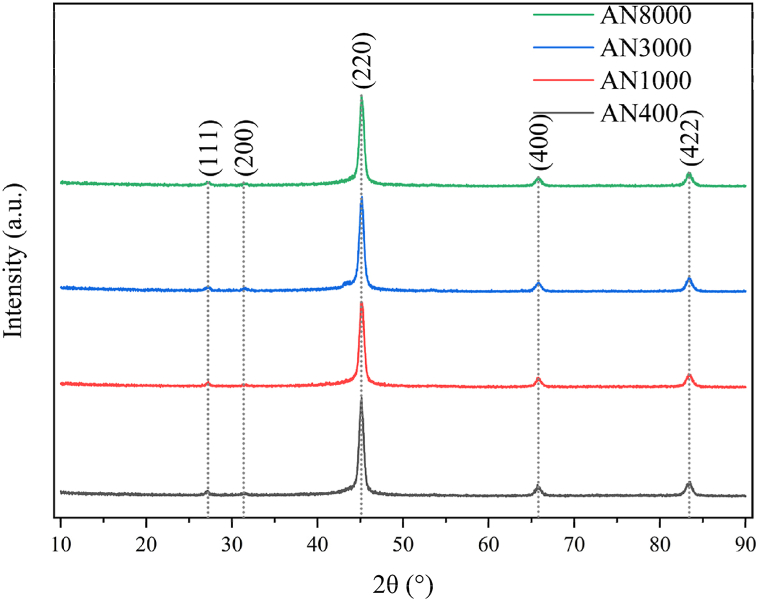
XRD patterns of Fe_58.5_Si_16.7_B_6.5_Nb_5.1_Cu_13.2_ nanocrystalline shielding films. Fe_3_Si phase peaks are detected, signifying the existence of precipitated Fe_3_Si phase post-annealing. Despite fragmentation, XRD curves exhibit consistent Fe_3_Si phase peaks, implying the retention of the nanocrystalline structure. The intensity of diffraction peaks remains relatively unchanged with increasing fragmentation, suggesting minimal influence on the Fe_3_Si phase content. In essence, the composition and content of the Fe_3_Si phase in the nanocrystalline alloy remain stable regardless of the degree of fragmentation.

Fig. 7 displays the XRD patterns of the chosen nanocrystalline alloy shielding films with varying degrees of fragmentation. The highest diffraction peaks observed correspond to the (220) plane of Fe_3_Si phase, indicating the presence of precipitated Fe_3_Si phase within the amorphous matrix following annealing treatment. Despite the fragmentation applied, the XRD curves consistently exhibit the Fe_3_Si phase at distinct magnetic permeability levels, showcasing an unaltered nanocrystalline structure. Furthermore, the intensity of the diffraction peaks remains relatively constant as the degree of fragmentation rises. This suggests that the content of the Fe_3_Si phase is minimally impacted by the fragmentation process. Essentially, the composition and quantity of the Fe_3_Si phase in the nanocrystalline alloy remain unchanged regardless of the fragmentation degree.

The grain size can be calculated using Debye-Scherrer formula:

(1) $$D_{hkl}=K\lambda /\beta \;\cos\;\theta$$

where *K* represents the Scherrer constant (0.89), ***λ*** is the wavelength of Cu *Ka* radiation (*λ* = 0.154056 nm), *β* is the full width at half maximum (FWHM) of the diffraction peak profile, and *θ* is the diffraction half-angle.

The grain size calculations presented in Table 3 reveal that the nanocrystalline alloy's grain sizes remain essentially unchanged at approximately 14 nm for different magnetic permeability samples. These findings suggest that the fragmentation process does not affect the grain size of the nanocrystalline alloy. This can be attributed to the fact that the fragmentation process primarily divides the nanocrystalline alloy into macroscopic block regions, leaving its microstructure unaffected.

**Table 3:** Grain size of nanocrystalline alloy at various fragmentation degrees.

Specimen number	*2θ* (°)	*β* (°)	Grain size (nm)
AN400	45.084	0.615	14
AN1000	45.132	0.609	14
AN3000	45.109	0.607	14
AN8000	45.123	0.728	14

### 3.2 Shielding properties of Fe_58.5_Si_16.7_B_6.5_Nb_5.1_Cu_13.2_ nanocrystals

The shielding properties of nanocrystalline films can be evaluated by examining their complex permeability (*μ*_*r*_ = *μ'* - *jμ"*) responses to alternating magnetic fields. Real component (*μ′*) reflects the ability to store magnetic energy, while imaginary part (*μ"*) indicates the capacity for energy dissipation.

**Fig. 8:**
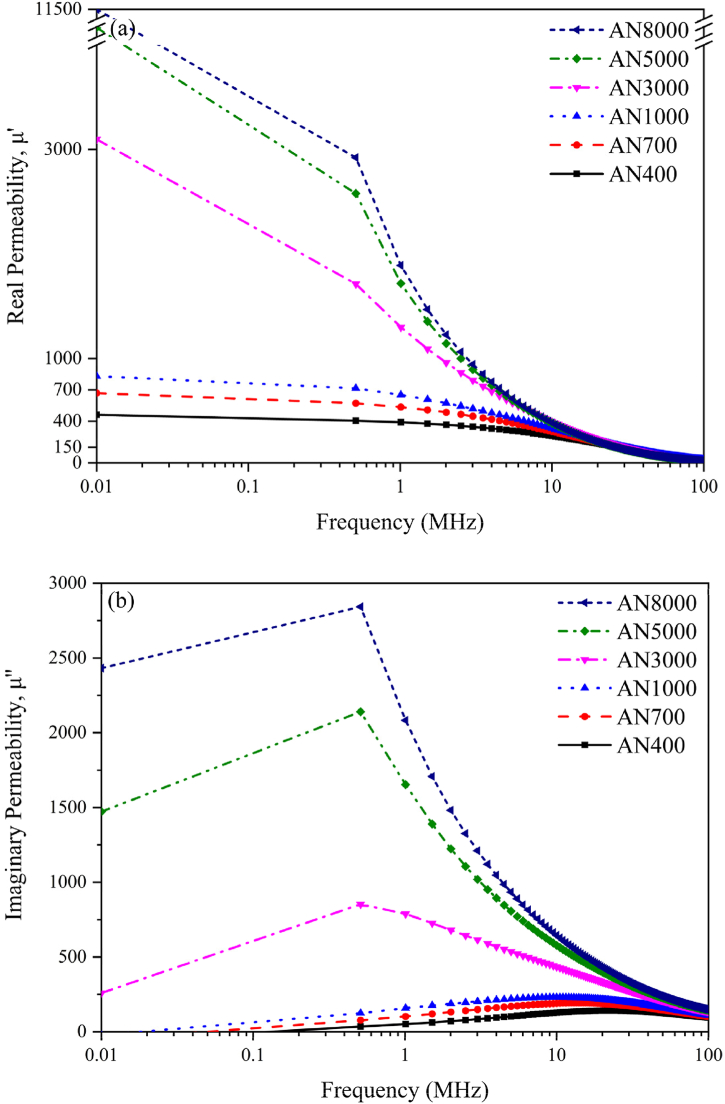
Frequency-dependent behavior of complex permeability for Fe_58.5_Si_16.7_B_6.5_Nb_5.1_Cu_13.2_ nanocrystalline alloy. In Fig. 8(a), *μ′* ranges from 400 to 11,500, whereas in Fig. 8(b), *μ"* ranges from 20 to 2800. Both *μ′* and *μ"* demonstrate substantial frequency dependence owning to eddy current and hysteresis losses. Higher operation frequencies result in reduced shielding efficacy due to heightened losses from eddy currents. The complex permeability is affected by both the degree of fragmentation and the soft magnetic characteristics of the alloy.

Fig. 8 illustrates the frequency-dependent behavior of complex permeability for nanocrystalline alloy specimens. In Fig. 8(a), *μ′* values range from 400 to 11,500, whereas in Fig. 8(b), *μ"* values range from 20 to 2800. Both *μ′* and *μ"* demonstrate substantial frequency dependence owning to eddy current and magnetic hysteresis losses. As the working frequency of the induction coil increases, the shielding properties of nanocrystalline alloy tend to diminish. This can be attributed to amplified losses caused by eddy current, which impede the penetration of magnetic field into the interior of the alloy. This phenomenon can be characterized by the decrease in *μ′* and *μ"*. Furthermore, as the degree of fragmentation intensifies, the average bulk size of the alloy decreases, and the gaps between fragments are filled with acrylic ester adhesive. This leads to an increase in magnetic resistance and a reduction in eddy current losses. The relationship between *μ''* and the degree of fragmentation follows an inverse pattern. Overall, the fragmentation process mitigates the impact of eddy current losses, and the magnitude of the complex permeability is influenced by both the degree of fragmentation and the soft magnetic properties of the alloy. The magnetic losses at high frequencies encompass eddy current loss, hysteresis loss, and residual loss. Given that nanocrystalline soft magnetic materials possess high permeability and low coercivity (<1.5A/m), the hysteresis loss for these materials is considered negligible. In low-frequency scenarios, such as those relevant to this induction heating application, residual loss is also attributed to hysteresis loss. Consequently, hysteresis loss and residual loss have not been taken into account.

In addition to complex permeability, the magnetic loss tangent angle (tan*δ*_*μ*_) is commonly utilized to access magnetic shielding properties. It is calculated as the ratio of *μʺ* to *μʹ* and can be expressed as follows ^[52]^:

(2) $$\tan\;\delta_{\mu }=\mu ʺ/\mu ʹ$$

The magnetic loss tangent angle represents the percentage of energy loss to energy stored during alternating magnetization. A higher absolute value of tan*δ*_*μ*_ indicates a greater susceptibility to magnetic losses.

**Fig. 9:**
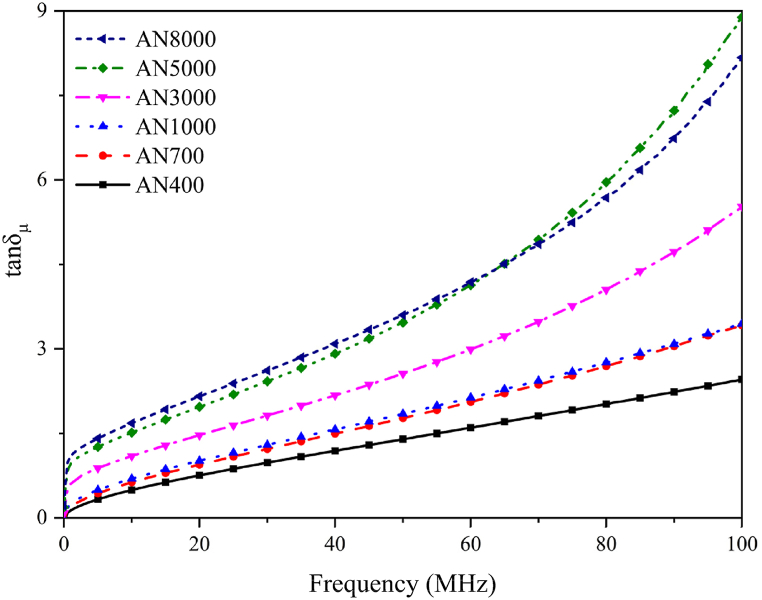
Frequency dependencies of tan*δ*_*μ*_ for Fe_58.5_Si_16.7_B_6.5_Nb_5.1_Cu_13.2_ nanocrystalline alloy. The tan*δ*_*μ*_ values range from 0 to 9.0, with larger absolute values signifying greater susceptibility to magnetic losses. Lower fragmentation degrees in nanocrystal shielding films are associated with higher tan*δ*_*μ*_ values, indicating elevated magnetic losses. This trend is linked to intensified eddy current effects in nanocrystalline alloys resulting from reduced fragmentation, hence amplifying magnetic losses.

Fig. 9 delineates the tan*δ*_*μ*_ curves, indicative of the magnetic loss factor, for Fe_58.5_Si_16.7_B_6.5_Nb_5.1_Cu_13.2_ nanocrystalline alloys, each variant distinguished by its unique magnetic permeability. The depicted curves encompass a tan*δ*_*μ*_ range from 0 to 9.0. Notably, an elevated tan*δ*_*μ*_ value is suggestive of a diminished level of microstructural fragmentation within the nanocrystalline alloy matrix. Such an increase in losses is attributable to the magnified influence of eddy current phenomena in less fragmented materials, thereby engendering a more pronounced dissipation of energy. Upon closer inspection of the curves, a pronounced upward trajectory is observed in the vicinity of 0 MHz at the inception of the line segments. This abrupt elevation is attributed to the incipient augmentation of magnetic energy dissipation, concurrent with the incremental rise in frequency from a state of fundamental inactivity. The pronounced slope of the curve marks the commencement of magnetic losses, which progressively assume greater prominence in accordance with the augmentation of the frequency spectrum.

Regarding the anomalous behavior wherein the tan*δ*_*μ*_ curve for AN5000 surpasses that of AN8000 post-60 MHz, this deviation can be elucidated through an examination of the intricate dynamics of loss mechanisms in nanocrystalline materials at elevated frequencies. As previously expounded upon, in the regime of high frequencies (approximately or exceeding 60 MHz), the loss profile of nanocrystalline materials becomes significantly more complex, incorporating hysteresis losses alongside residual losses. It is plausible for tan*δ*_*μ*_ curves, each representing alloys of disparate permeability, to intersect at particular frequencies. This phenomenon is attributable to the superposition of residual loss mechanisms that become operative under these frequencies.

The parameter *R*_*S*_ serves as a metric for quantifying the power output of a heating element when subjected to a constant induction heating current. Its value is contingent upon several factors including the characteristics of the coil, the intrinsic properties of the heating element itself, the aluminum shell, and the shielding film. The operational principle of an induction heating apparatus hinges on the generation of alternating magnetic fields, which in turn induce eddy currents within the heating element. The dissipation of these induced currents as joule heat constitutes the primary mechanism by which material heating is achieved.

**Fig. 10:**
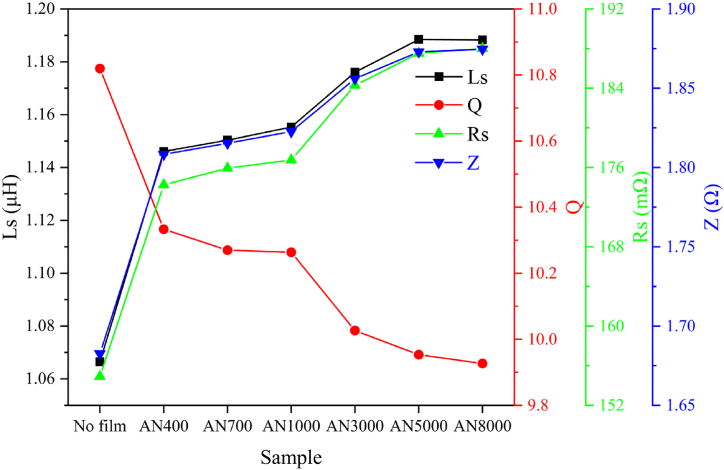
Impedance Parameters (*L*_*S*_, *R*_*S*_, *Q* and *Z*) of an induction heating unit under single-turned nanocrystalline alloy as shielding film. *R*_*S*_ reflects the power level of the heater under a consistent induction heating current. A higher magnetic permeability leads to a 10 % increase in *Rs*. *L*_*S*_ and *Z* exhibit the same trend as *R*_*S*_.

Fig. 10 illustrates the changes in impedance parameters (*L*_*S*_, *R*_*S*_, *Q* and *Z*) for an induction heating unit with various single-turned magnetic shielding films composed of 3-layered nanocrystalline alloy ribbons. *R*_*S*_ reflects the power level of the heater under a consistent induction heating current. A higher magnetic permeability leads to a 10 % increase in *Rs*. *L*_*S*_ and *Z* exhibit the same trend as *R*_*S*_. Initially, augmenting magnetic permeability enhances *R*_*S*_, *L*_*S*_, and *Z*; however, this impact diminishes with higher permeability. This indicates that the heat efficiency in the heating device improves with higher *μʹ* (magnetic permeability of the magnetic shielding film), eventually stabilizing at a constant level.

**Fig. 11:**
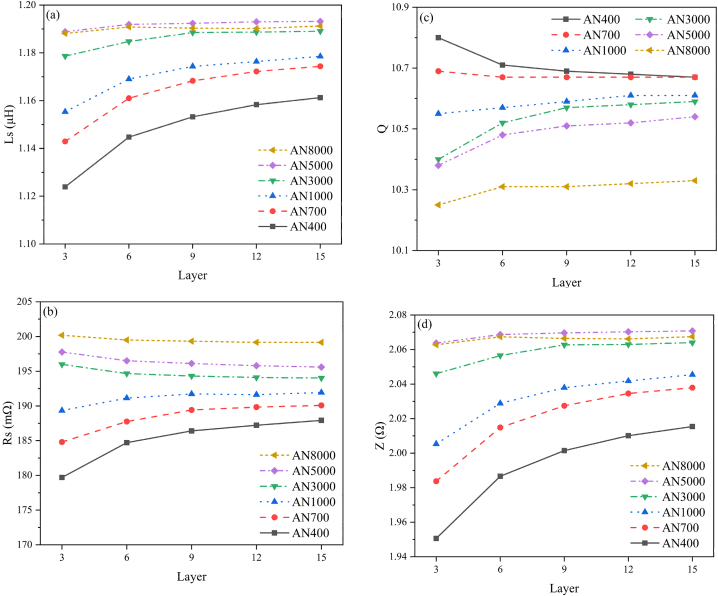
Variation of *L*_*S*_ (a), *R*_*S*_ (b), *Q* (c) and *Z* (d) of an induction heating unit with varying numbers of nanocrystalline ribbon layers (3–15 layers). The data reveals that *R*_*S*_ and *Q* values for samples AN8000, AN5000, and AN3000 decrease as the layer count increases, reaching a peak at 3 layers, whereas other samples exhibit an opposite trend. Higher *R*_*S*_ values indicate a greater potential for heat conversion within the induction system. For this study, 9 layers (3 turns) of shielding film were selected, which may not be optimal for high magnetic permeability shielding films but are advantageous for those with low magnetic permeability. This choice does not influence the overall trend observed in heating efficiency.

Fig. 11 illustrates the correlation between the numbers of nanocrystalline ribbon layers (Layers = Turns * 3) and the *L*_*S*_, *R*_*S*_, *Q* and *Z* parameters of an induction heating unit. As the layer count increases, there is a notable impact on the heating performance of the induction system, as indicated by the slope approaching zero. Higher *Rs* values indicate enhanced heat conversion potential within the induction heating system. However, the integration of magnetic shielding films up to 5 turns gradually stabilizes *Rs*, indicating a reduced impact on the heating element. Owning to the limitations imposed by device thickness, the analysis of energy consumption in induction heating is confined to 9 layers, corresponding to 3 turns. It's pertinent to note that LCR measurements are performed on the coil in isolation from the device casing, thereby negating any constraints related to size; in this configuration, the coil is capable of accommodating up to 5 turns or more. However, when evaluating the total energy consumption of the device, the tests are conducted with the device casing included. The dimensions of the casing determine that the shielding film can only be wrapped for 4 turns, achieving a total of 12 layers, at which point it nearly touches the inner wall of the casing, thus precluding additional turns. Furthermore, wrapping the shielding film for 4 turns results in portions of the film being in close proximity to the casing, thereby causing conductive energy losses between the two. To mitigate the impact of these conductive losses, the tests for total device power consumption were executed using 3 turns, equivalent to 9 layers.

It is important to note that in Fig. 11, the *R*_*S*_ and *Q* values for samples AN8000, AN5000, and AN3000 decrease with an increasing number of layers, peaking at 3 layers, while other samples show an increase. In the context of this study, nanocrystalline shielding films with higher magnetic permeability tend to incur greater losses, whereas those with lower magnetic permeability do not achieve optimal shielding effectiveness at 3 layers. Increasing the number of layers can further enhance shielding performance. Additionally, *R*_*S*_ and *Q*, which are often associated with the power level of the entire induction heating system including the coil, shielding film, and heat generator, do not directly represent heating efficiency. These values were measured under low current levels using an LCR meter. The actual heating efficiency in practical applications is determined by measuring the power consumption of the power source. Therefore, *R*_*S*_ and *Q* serve only as a reference and are not the sole criterion for evaluation.

In selecting the number of layers for the shielding film in this application, a layer value of 9 (3 turns) is chosen. This might be suboptimal for high magnetic permeability shielding films but favorable for low magnetic permeability ones. When tested in practical applications, this did not affect the trends regarding heating efficiency.

**Fig. 12:**
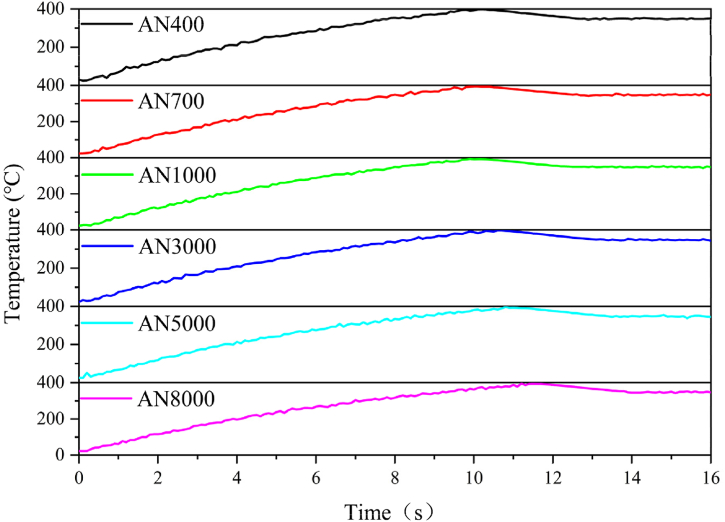
Preheating temperature rise curves for an induction heating device with 12-layered nanocrystalline shielding films. The time needed for preheating from room temperature to 400 °C exhibits a slight initial decrease with rising magnetic permeability, succeeded by an upward trajectory. The minimum preheating time of 9.9 s to reach 400 °C is achieved when *μʹ* equals 1000.

The temperature rise curves during the ramp-up to 400 °C for an induction heating device equipped with 12-layered nanocrystalline shielding films are illustrated in Fig. 12. The time needed for preheating from room temperature to 400 °C exhibits a slight initial decrease with rising magnetic permeability, succeeded by an upward trajectory. The minimum preheating time of 9.9 s to reach 400 °C is achieved when μʹ = 1000.

**Fig. 13:**
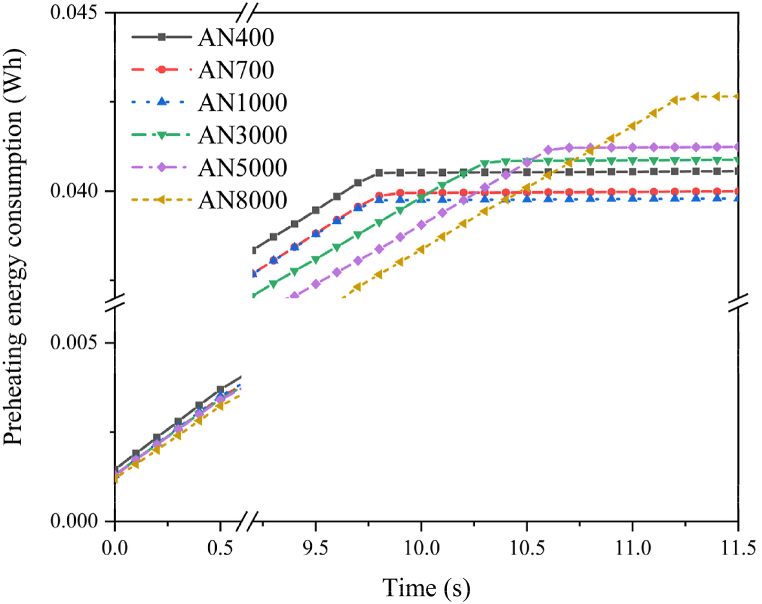
Preheating energy consumption curves for an induction heating device featuring 12-layered nanocrystalline shielding films. The graph illustrates the correlation between permeability (*μʹ*) and preheating energy consumption for 4-turned nanocrystalline alloy films during the preheating process up to 400 °C. The data indicates an initial decrease in energy consumption with increasing permeability, followed by a subsequent rise. Remarkably, at a permeability of *μʹ* = 1,000, the preheating energy consumption reaches its lowest value.

In Fig. 13, the graph represents the heating energy consumption curves for 4-turned nanocrystalline alloy films during the preheating process up to 400 °C. It is evident that with increasing the permeability, there is an initial decrease in preheating energy consumption followed by an increase. At a permeability of *μʹ* = 1,000, the preheating energy consumption is minimized at 0.03975 Wh. This result is primarily attributed to the higher permeability in the shielding material, leading to an increased *R*_*s*_ value which indicates greater eddy current conversion in the heating element. However, higher permeability also reduces the fragmentation degree, causing more significant eddy current losses within the shielding material itself, thereby accelerating energy dissipation. Consequently, the 4-turned nanocrystalline alloy films with a permeability of *μʹ* = 1000 demonstrate the highest efficiency for this induction heating application and offer improved shielding effectiveness.

**Fig. 14:**
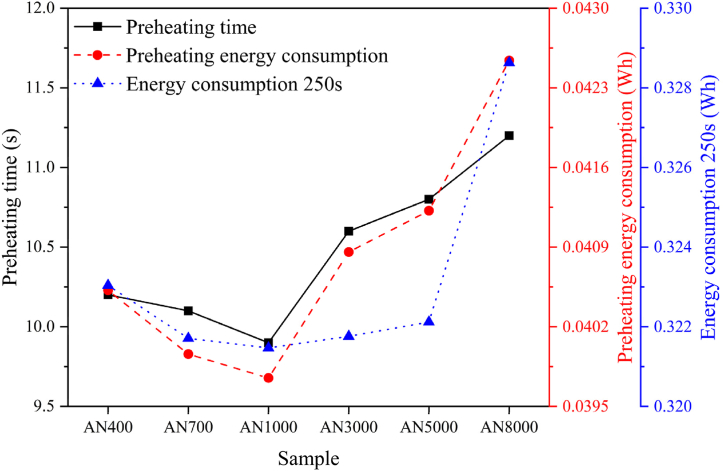
Comparative analysis of preheating time and energy consumption in 12-layered nanocrystalline shielding films with varying permeability for an induction heating device. These curves exhibit a gradual downward trend as permeability increases, signifying enhanced shielding efficiency within a specific range. However, a continuous rise in permeability leads to an upward trajectory. The AN1000 sample showcases the shortest preheating time, lowest preheating energy consumption, and minimal energy consumption after reaching 400 °C for 250 s. Consequently, the 12-layered nanocrystalline shielding film with *μʹ* = 1000 demonstrates the exceptional shielding effectiveness for this induction heating application, resulting in a 12.5 % reduction in preheat time and 7 % less energy consumption during preheating.

Fig. 14 presents the comparative analysis of preheating time, preheating energy consumption, and energy usage over 250 s after reaching 400 °C in 12-layered nanocrystalline shielding films with varying permeability for an induction heating device. The depicted curves exhibit a gradual downward trend as permeability increases, signifying enhanced shielding efficiency within a specific range. However, a continuous rise in permeability leads to an upward trajectory. Notably, the AN1000 sample showcases the shortest preheating time, the lowest preheating energy consumption, and the minimal overall energy consumption upon achieving 400 °C for 250 s. Consequently, the 12-layered nanocrystalline shielding film with a permeability of *μʹ* = 1000 demonstrates the exceptional shielding efficiency for this induction heating application, resulting in a 12.5 % reduction in preheat time and 7 % less energy consumption during preheating.

**Fig. 15:**
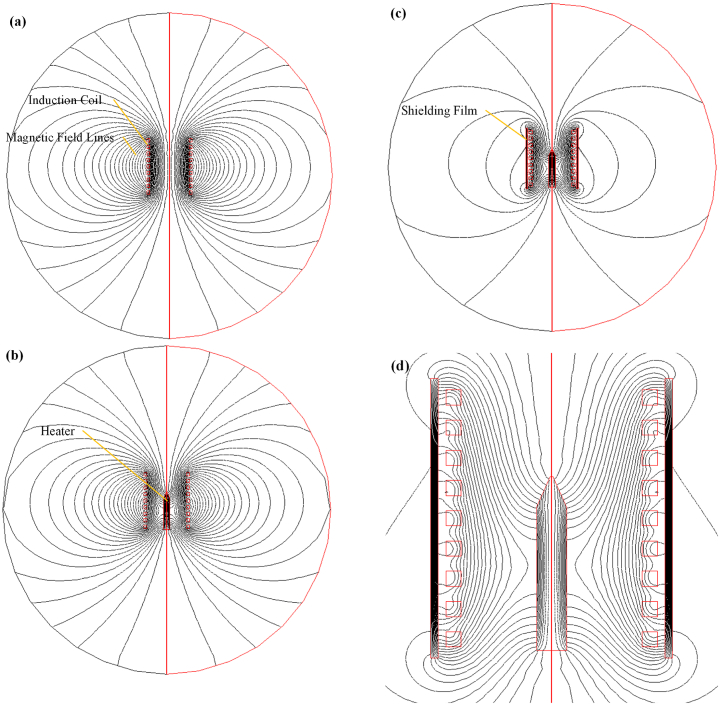
Schematic diagrams depicting spatial distribution of magnetic fields in an induction heating system: (a) Induction coil only, (b) With heater, (c) With shielding film and heater, (d) Detailed magnetic field lines with shielding film and heater. In Fig. 15(a), the coil produces magnetic field lines that form closed loops, with higher density near the coil. Fig. 15(b) introduces a high-permeability heater that absorbs a majority of the magnetic field lines, converting them into heat. Fig. 15(c) depicts the incorporation of a shielding film wrapped around the coil, causing external magnetic field lines to concentrate on it, thereby amplifying the flux density within the coil. Fig. 15(d) offers a detailed view of the interaction between magnetic field lines and the shielding film. The presence of the shielding film optimizes the distribution of magnetic fields, improving heating efficiency within the induction coil.

Fig. 15 portrays the schematic diagrams depicting spatial distribution of magnetic fields in an induction heating system under various conditions. In Fig. 15(a), the system comprises solely the induction coil without a heater or shielding film. The coil generates magnetic field lines forming closed loops around it, with higher density near the coil due to concentrated magnetic flux. Notably, the magnetic field strength is sparser in the center of the coil compared to its vicinity. A similar model has been proposed in a patent application by Philip Morris Products S.A [31]. However, this model did not account for a heater within the coil, and the shielding mechanism was insufficiently elucidated. In Fig. 15(b), a needle-shaped soft magnetic alloy acts as the heater within the coil. When alternating currents pass through the coil, the heater absorbs most magnetic field lines due to its higher permeability. This absorption leads to heat generation through eddy current loss and hysteresis loss, facilitating efficient magnetic induction heating. External to the coil, the magnetic field lines do not participate in the heating process during a single-frequency cycle. However, if these field lines are not adequately shielded, they might interact with external conductive materials, leading to eddy current losses and reduced electrical energy efficiency. In Fig. 15(c), a shielding film is introduced, encircling the outer surface of the coil. Fig. 15(d) offers a detailed view of the interaction between magnetic field lines and the shielding film. The shielding film aims to adjust the magnetic field distribution outside the coil. It enables magnetic field lines originating from the coil to focus on the film, creating closed loops. This configuration boosts the magnetic flux density within the coil, enhancing energy efficiency during induction heating. This improvement is primarily due to the shielding film's higher permeability relative to air. Magnetic field lines tend to follow paths with lower impedance, and the shielding film's elevated permeability provides a more favorable route for magnetic flux. Consequently, the shielding film optimizes the magnetic field line distribution, enhancing heating efficiency within the induction coil.

**Fig. 16:**
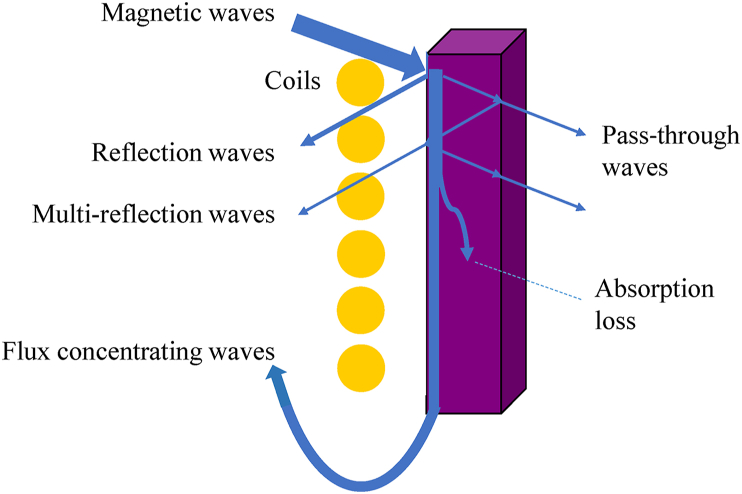
Theoretical model illustrating shielding mechanism in an induction heating system. The shielding film guides the majority of magnetic field generated by the spiral coils to pass through it, creating loops with internal waves and reflecting a fraction as reflected waves. Various wave types entering the film include flux concentrating waves, pass-through waves, and absorption loss. By incorporating the shielding film, magnetic induction intensity dramatically rises as it steers magnetic field lines and bolsters flux density within the coil. Total magnetic flux intensity (*B*_*T*_) is represented as the sum of magnetic flux density within the shielding film (*B*_*F*_), surface reflection waves (*B*_*R*_), multi-reflection waves (*B*_*MR*_), pass-through waves (*B*_*PT*_), and absorption loss (*B*_*AL*_).

The shielding film plays a crucial role in improving the efficiency of induction heating systems. A theoretical model has been proposed to elucidate its operational mechanism, as depicted in Fig. 16. The shielding film efficiently channels the majority of the magnetic field produced by the spiral coils, guiding them to flow through and intertwine with the internal waves to create loops, while a minor portion is reflected. The waves that penetrate the film can be classified into flux-concentrating waves, pass-through waves, and absorption losses.

By incorporating the shielding film, there is a substantial augmentation in the magnetic induction intensity due to the effective guidance of magnetic field lines and the heightened flux density within the coil. The total magnetic flux intensity (*B*_*T*_) originating from the induction coil is represented as the sum of various components, including the magnetic flux density within the shielding film (*B*_*F*_), surface reflection waves (*B*_*R*_), multi-reflection waves (*B*_*MR*_), pass-through waves (*B*_*PT*_), and absorption losses (*B*_*AL*_), as detailed in Equation (3).

(3) $$B_{T}=B_{F}+B_{R}+B_{MR}+B_{PT}+B_{AL}$$

The shielding efficiency (η) of the film is defined as the ratio of (B_F_ + B_R_ + B_MR_) to the total magnetic flux intensity (*B*_*T*_), as expressed in Equation (4).

(4) $$\eta =\left(B_{F}+B_{R}+B_{\text{MR}}\right)\;/\;B_{T}$$

where *B*_*F*_ signifies the film's ability to confine magnetic flux and is dependent on the real part of permeability (*μ′*) and the number of layers or thickness for nanocrystalline shielding films. The contributions of *B*_*R*_ and *B*_*MR*_, which indicate the film's capacity to reflect magnetic field lines, are considered negligible. *B*_*AL*_ reflects the film's capacity to absorb magnetic field lines, encompassing factors like magnetic loss (tan*δ*_*μ*_), resistive loss, and dielectric loss, influenced by parameters such as *μ′*, *μ”*, conductivity, current density, working frequency, and temperature. Given that numerous magnetic field lines transverse the shielding film and interact with it, the impact of *B*_*AL*_ cannot be disregarded.

Further research is essential to investigate the specific values and proportions of parameters such as *B*_*F*_，*B*_*AL*_ and *η*. The comprehension and enhancement of magnetic shielding film performance hinge on these parameters. Subsequent analysis can delve into discerning the specific values under various experimental circumstances and establishing their correlations. This exploration will offer insights into the interplay among magnetic field confinement, absorption loss, and overall shielding efficiency, guiding the design and enhancement of shielding films for induction heating systems.

The theoretical model illustrates previous experimental data. Initially, augmenting the permeability of the shielding film bolsters its flux concentration capability (*B*_*F*_) and amplifies shielding efficiency. Nevertheless, further escalation leads to reduced fragmentation, heightened overall conductivity, and increased eddy current loss, contributing to *B*_*AL*_. This elucidates the identified yielding point at *μʹ* = 1000 for this scenario. Moreover, the quantity of nanocrystalline layers in the film also impacts shielding efficiency. Initially, increasing layer count enhances *B*_*F*_ and diminishes pass-through waves (thus lowering *B*_*AL*_), thereby refining shielding efficiency. However, additional increases do not notably boost shielding efficiency since pass-through waves are already substantially mitigated.

This theoretical model offers critical insights into interpreting experimental outcomes and leads support to the development of shielding films for forthcoming induction heating systems. Its application is pivotal for fine-tuning magnetic shielding performance and advancing the technological capabilities of induction heating systems.

## 4 Conclusions

In this study, a comprehensive investigation was conducted on iron-based (Fe_58.5_Si_16.7_B_6.5_Nb_5.1_Cu_13.2_) nanocrystalline shielding films to gain insights into their material characteristics, magnetic properties, and magnetic shielding performance, particularly for induction heating applications. The principle findings and conclusions can be summarized as follows.

1) Increasing the fragmentation degree in Fe_58.5_Si_16.7_B_6.5_Nb_5.1_Cu_13.2_ nanocrystalline shielding films results in a simultaneously reduction in both the real and imaginary parts of permeability, leading to a weakened magnetic loss property. This fragmentation induces air gaps within the nanocrystalline shielding films, effectively reducing eddy current loss.
2) The heat conversion efficiency of induction heating device exhibits an upward trend with increasing μʹ (real part of permeability) of magnetic shielding films but reaches a plateau beyond a certain threshold. Similarly, increasing the number of the nanocrystalline alloy layers enhances the shielding effect of the induction heating device; however, the improvements become negligible beyond 12 layers.
3) As magnetic permeability escalates, the preheating time from room temperature to 400 °C, preheating energy consumption, and energy consumption for a duration of 250 s after reaching 400 °C initially decrease before subsequently increasing. The exceptional shielding effect is achieved when μ′ reaches a value of 1000.
4) The theoretical model developed in this study offers a comprehensive understanding of the factors influencing shielding efficiency of nanocrystalline shielding films. It effectively explains the observed trends in magnetic permeability, magnetic loss, and magnetic shielding performance, supporting the practical use of these films in induction heating systems.

By integrating the theoretical model with experimental findings, this study provides a comprehensive understanding of the behavior of Fe_58.5_Si_16.7_B_6.5_Nb_5.1_Cu_13.2_ nanocrystalline shielding films. These insights have important implications for the advancement of nanocrystalline materials in magnetic shielding for induction heating applications. Further research is required to investigate specific parameter values and their interrelationships, facilitating the design and enhancement of shielding films to improve induction heating system efficiency.

## Funding information

This research was funded by the Major Science & Technology Project of 10.13039/501100008862China National Tobacco Corporation (No.110202101073 (XX-18)), and the Standard Project of 10.13039/501100008862China National Tobacco Corporation (No. 2020QB005).

## Prime novelty statement

1. This study unveils the impact of fragmentation degree in nanocrystalline Fe-based alloys on magnetic losses, preheating time, and power consumption in high-frequency induction heating systems.
2. The research demonstrates that increased fragmentation degree leads to reduced absorption losses by decreasing both real and imaginary components of magnetic permeability.
3. The findings highlight the significance of achieving a relative permeability value of 1000 for optimal electromagnetic shielding performance in nanocrystalline shielding films.
4. Additionally, the study develops a theoretical model elucidating the shielding mechanism, offering valuable insights for the effective application of nanocrystalline shielding materials in induction heating systems.

## CRediT authorship contribution statement

**Feng Li:** Formal analysis, Conceptualization. **Ruifeng Zhao:** Investigation, Data curation. **Yibo Liu:** Validation, Investigation. **Yang Xiao:** Writing – original draft, Data curation. **Peng Sun:** Methodology, Data curation. **Jiamao Luo:** Visualization, Validation. **Jun Wen:** Resources. **Zhihong Chen:** Resources. **Jing Hu:** Supervision, Project administration, Conceptualization. **Zuqiang Qi:** Writing – review & editing, Supervision, Formal analysis.

## Declaration of competing interest

The authors declare that they have no known competing financial interests or personal relationships that could have appeared to influence the work reported in this paper.
